# The amplitude of low-frequency fluctuations is correlated with birth trauma in patients with postpartum post-traumatic stress disorder

**DOI:** 10.1038/s41398-024-03018-3

**Published:** 2024-08-14

**Authors:** Chunlian Chen, Bo Li, Liping Chai, Kai Liu, Shufen Zhang

**Affiliations:** 1 Jinzhou Medical University, Jinzhou, Liaoning China; 2 Department of Radiology, The 960th Hospital of the PLA Joint Logistic Support Force, Jinan, Shandong China; 3 Department of Obstetrics, Shandong Second Provincial General Hospital, Jinan, Shandong China

**Keywords:** Pathogenesis, Psychiatric disorders

## Abstract

Postpartum post-traumatic stress disorder (PP-PTSD) is a severe mental disorder worldwide. In recent years, some studies have reported that PP-PTSD stems from birth trauma. The present study was dedicated in finding ways to predict the occurrence of emergency caesarean section (ECS), trying to analyze the methods to reduce incidence of PP-PTSD on this basis, further exploring the neuroimaging changes in PP-PTSD. A total of 245 primiparas with intention of vaginal delivery were recruited. The internal tocodynamometry measurement was performed during labor for all mothers, and respectively taken at 3–5 cm, 5–8 cm, and 8–10 cm of cervical dilation. The receiver operating characteristic (ROC) curve and Binary logistic regression analyses were also performed to identify fetal head descending thrust that might help in the prediction of ECS. Resting-state magnetic resonance imaging (MRI) was performed on 26 patients diagnosed with PP-PTSD of 245 mothers, the amplitude of low-frequency fluctuations (ALFF) technology was used to observe the spontaneous neural activity of all PP-PTSD patients and correlation analyses were performed. We found that the natural delivery rate of mothers with fetal head descending thrust <16.29 N (5–8 cm), 26.36 N (8–10 cm) were respectively lower than other mothers with fetal head descending thrust ≥16.29 N (5–8 cm), 26.36 N (8–10 cm) (*P* < 0.05). The ROC curve analysis showed that the area under the receiver operating characteristic curve (AUC) value of thrust (5–8 cm) was 0.896 (95% CI: 0.854–0.938, *p* < 0.001), AUC of thrust(8–10 cm) was 0.786 (95% CI: 0.714–0.858, *p* < 0.001), which showed strong potential for predicting ECS. In addition, the Binary logistic regression analysis showed thrust (5-8 cm) and thrust (8–10 cm) were independent correlates of ECS. The resting-state functional magnetic resonance imaging (rs-fMRI) results indicated that PP-PTSD group showed decreased ALFF in the bilateral insula cortex (IC), right anterior cingulate cortex (ACC), and left midcingulate cortex (MCC) compared with healthy postpartum women (HPW) (false discovery rate (FDR) correction *q*-value < 0.05). The ALFF value of the right ACC was positively correlated with the Perinatal Post-traumatic stress disorder Questionnaire (PPQ) score (r = 0.4046 *p* = 0.0403) and Posttraumatic Stress Disorder Checklist–Civilian Version (PCL–C) score (r = 0.3909 *p* = 0.0483). The internal tocodynamometry measurement can serve as a predictive tool for ECS, on this basis, the implementation of effective emotional support may help to reduce the incidence of PP-PTSD. Besides, this study has verified the presence of altered ALFF in the brain regions of PP-PTSD patients, mainly involving the bilateral IC, right ACC, and left MCC, that might be associated with emotion, cognition, and memory disorders functions in PP-PTSD patients.

## Introduction

Postpartum post-traumatic stress disorder (PP-PTSD), one of the common mental disorders occurring during the postpartum period, which is recognized as a potential consequence of a series of birth trauma events [1]. The main characteristics of PP-PTSD included intrusion (re-experiencing the feelings of trauma), avoidance (avoiding issues associated with birth and infants), staying at a high status of alertness, negative cognitive and emotional experiences according to the Diagnostic and Statistical Manual of Mental Disorder (DSM-5) [2]. It is estimated that the rates of PP-PTSD is high, with a global prevalence of around 20% of women [3, 4]. PP-PTSD due to birth trauma can affect maternal wellbeing as well as the mother–child relationship and partner relationship [5]. Childbirth is generally regarded as a positive experience. However, 19.7–45.0% of women may consider childbirth as a traumatic and distressing event in their lives [3]. Birth trauma is a complex concept which is used to describe a series of related experiences of childbirth, and negative psychological responses to childbirth [6]. Birth trauma refers to the circumstances of childbirth and delivery that cause actual, potential serious injury or death to the mother and baby, causing the mother to experience intense fear, loss of control and helplessness [7]. Trigger of birth trauma experiences include labor pain, anxiety before childbirth, concern for the newborn child, pre-existing depression, and lack of support during childbirth [8]. Another important factor seems to be the birth mode and unplanned interventions in emergency situations, among them, emergency cesarean section (ECS) is the most traumatizing and associate with more PP-PTSD symptoms compared to other birth modes [8, 9]. Actually, more than 1/3 of mothers experienced their delivery as a traumatic event, while 1/4 of them would experience PP-PTSD [4]. Women with PP-PTSD usually present with re-experimentation of the event, a feeling of disconnection from the baby, absence of reality, nightmares, irritability, rejection of new motherhood, or may even develop tokophobia [10–13]. These symptoms most often appear between 4-6 weeks postpartum, and remain for months or years later, and even in future pregnancies [13–15]. Pregnancy, childbirth, and postpartum are periods in which a woman’s risk of developing mental disorder increases, increasing social attention to mother’s health due to the high incidence and severe impact. It is vital for devel oping more accurate preventions and interventions, reducing the toll of birth trauma on mothers to decrease reducing morbidity of PP-PTSD, resulting in better results for the mother and baby well-being.

A coordinated series of uterine contractions is necessary for vaginal delivery [10], labor is caused by contracting the uterine muscles pushing the fetus out [16]. The existed mild uterine contractions throughout gestation become gradually more frequent and intense, transitioning at some point to labor process, when the uterine forces dilate the cervix and propel the fetus down the birth canal. The obstetrician-gynecologists judge labor primarily by the pattern of changes in cervical dilatation and fetal descent, and by other observations, such as the frequency and quality of uterine contractions. Four available clinical approaches are frequently assessed uterine contraction, including manual palpation, intrauterine pressure determination, tocodynamometry and electrohysterography [10]. Particularly, the monitoring of uterine contractions by internal tocodynamometry during labor is advocated by professional societies in obstetrics and gynecology, because of its greater accuracy than other approaches [17]. In this study, internal tocodynamometry measurement was used to measure fetal head descending thrust to assess evaluation of labor process.

Functional magnetic resonance imaging (fMRI) is a non-invasive and refined temporal-spatial resolution technique, can reflect the spontaneous neural activity of brain by detecting the fluctuations in blood oxygenation level dependent (BOLD) signals [18]. Recently, resting-state fMRI (rs-fMRI) has been used to detect abnormal neural activity in a wide range of neurological or psychiatric diseases, such as major depressive disorder [19], bipolar disorder [20], postpartum depression [21] and post-traumatic stress disorder (PTSD) [22]. These researches have shown that rs-fMRI has been a sensitive and special technology for studying the alterations of brain function. The amplitude of the low-frequency fluctuation (ALFF) approach can use voxel-based analysis and focus on regions of spontaneous activity in the whole brain, directly reflecting the intensity of spontaneous neural activity at the baseline state [23]. Currently, no fMRI studies on PP-PTSD have been reported, so it is important to explore the abnormal activity of brain function in PP-PTSD patients.

In this study, we have two hypotheses: (1) Firstly, internal tocodynamometry measurement of all mothers are assessed during labor process. We hypothesize that ECS is necessary for some mothers who are unable to undergo vaginal delivery, their values of fetal head descending thrust may be different from other vaginal delivery mothers. These different values may be used as critical indicators for judging and predicting ECS, leaving more psychological and surgical preparation time for mothers and medical staff, and reducing the harm of birth trauma, thus decrease the risk of PP-PTSD for mothers. (2) secondly, we hypothesize that PP-PTSD patients exist the significant differences of the spontaneous brain activity compare with healthy postpartum women (HPW), which may serve as an important neuroimaging evidence for explicating the intricate neural mechanism of PP-PTSD.

## Materials and methods

### Participants

**Fig. 1:**
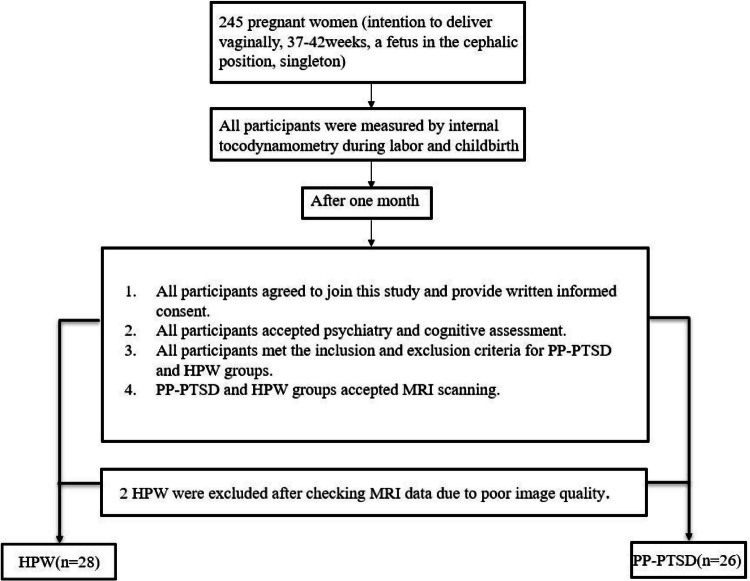
Postpartum post-traumatic stress disorder screening and study enrollment flow diagram. PP-PTSD postpartum post-traumatic stress disorder, HPW healthy postpartum women.

From 1 June 2020 to 1 January 2023, a total of 245 late trimester of pregnant women (intention of vaginal delivery, 37–42weeks, primipara women, a fetus in the cephalic position, singleton) were recruited (in flowchart, Fig. 1) at the Department of Obstetrics of the 960th Hospital of the PLA Joint Logistics Support Force and the Department of Obstetrics of Shandong Second Provincial General Hospital. The internal tocodynamometry measurement of all mothers were assessed during labor process. After one month delivery, these mothers needed to finish questionnaires of personal information and assessment scales, including the Perinatal Post-traumatic stress disorder Questionnaire (PPQ), Post-traumatic Stress Disorder Checklist–Civilian Version (PCL-C), the Edinburgh Postnatal Depression Scale (EPDS), Pittsburgh sleep quality index (PSQI) scales. We obtained written informed consent and all participants agreed to accept psychiatry and cognitive assessment, MRI scanning. This study was approved by the ethical committee of the Shandong Second Provincial General Hospital (XYK20211115) and the 960th Hospital of the PLA Joint Logistics Support Force (2020-25).

Two experienced senior psychiatrists diagnosed 26 PP-PTSD patients from 245 participants, using the Structured Clinical Interview for Diagnostic and Statistical Manual of Mental Disorders, Fifth Edition (DSM-V) and Chinese Classification and Diagnostic Criteria of Mental Disorders 3rd edition (CCMD-3). Further inclusion criteria for the PP-PTSD group were as follows: (a) ages ≥20 years, in the 1st month after delivery (healthy full-term infants), (b) the first onset without any treatment, (c) PPQ ≥ 19, PCL–C ≥ 38, (d) an indication for labor with intravenous oxytocin were eligible for the trial, (f) qualified prenatal and delivery assessment, (g) normal social support during labor and birth. The exclusion criteria were the following: (a) previous psychiatric problems, history of PTSD and trauma (particularly interpersonal violence), or first-degree relatives had psychiatric disorders, (b) negative experiences and severe fear of childbirth, subjective distress, previous abortion, psychological difficulties in pregnancy (particularly depression in early pregnancy), (c) substance abuse or dependence, positive results on serologic tests for human immunodeficiency virus or hepatitis B virus, (d) pregnancy complications, traumatic birth events other than emergency cesarean section, (e) women with uterine scar, prior miscarriage or pregnancy losses, (f) signs of an intrauterine infection or fetal distress, prematurity, or low-birth weight infants, (g)History of head trauma and intracranial tumor, (h) poor imaging quality or head motion, (i) organic abnormalities for MRI routine series.

30 healthy postpartum women were recruited into the HPW group and matched with the PP-PTSD group. 2 participants in HPW group were excluded after checking MRI data due to poor image quality. The inclusion criteria for the HPW group were as follows: (a) ages ≥20 years, in the 1st month after delivery (healthy full-term infants), (b) PPQ and PCL-C scores were within the normal range, (c) an indication for labor with intravenous oxytocin were eligible for the trial, (d) qualified prenatal and delivery assessment, (e) normal social support during labor. The exclusive criteria were the same as those in the PP-PTSD group.

### The internal tocodynamometry measurement

The internal tocodynamometry measurement was performed by using digital delivery monitor during the first stage of labor. Every participant was placed in a flat supine position, the midwifes connected the probe rod with the digital push tension meter, opened the digital push tension meter and connected the computer, then put a new condom on the digital push tension meter probe rod. In the interval period of contraction, the front part of the probe rod was slowly probed into the vagina and the front end of the probe rod touched the fetal head. In the interval period of contraction, the front end of the probe rod only touched the fetal head, and the detection starting point was when the force was 0 N. The downward thrust of fetal head descending thrust was monitored throughout contraction period, so that the longitudinal geometric axis of the probe rod was horizontally inserted into the vagina and remained unchanged throughout the monitoring process. During contractions, the midwife presses the outer shell with her hand to keep it in place and prevent it from moving relative to the bed surface, thus monitoring the entire contraction period.

**Fig. 2:**
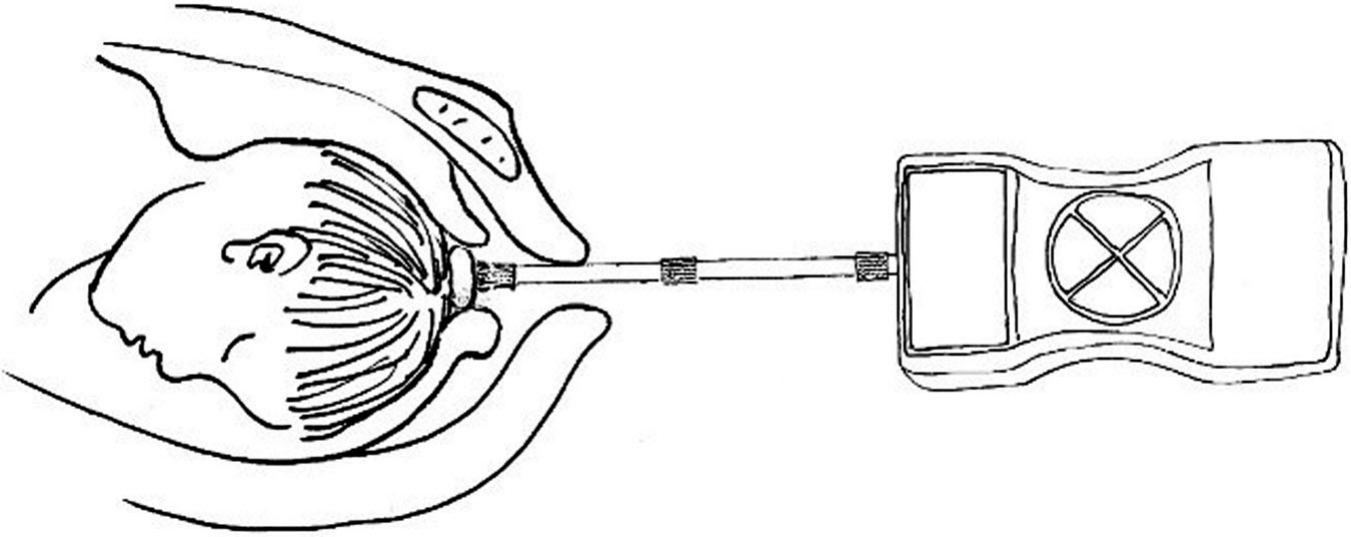
Schematic diagram of self-developed internal tocodynamometry machine. The internal tocodynamometry measurement was performed during labor for all mothers and respectively taken at 3–5 cm, 5–8 cm, and 8–10 cm of cervical dilation.

During the first stage of labor, when the cervix was dilated to 3 cm, the membranes were ruptured artificially and fetal head descending thrust was monitored. When the cervix was dilated to 3–5 cm, monitoring was performed every 60 minutes; when the cervix was dilated to 5–8 cm, monitoring was performed every 40 minutes; when the cervix was dilated to 8–10 cm, monitoring was performed every 20 minutes. Three complete cycles of contractions were monitored for each measurement, and recorded the average value respectively. A new condom was required for each measurement (Fig. 2).

### Clinical cognition assessments

PP-PTSD was diagnosed by PPQ and PCL-C. In addition, postpartum depression was assessed by using EPDS, sleep quality was assessed by using PSQI.

### MRI imaging acquisition

All MRI data were acquired on a 3.0 T MR system (Discovery MR750, General Electric, Milwaukee, WI, the United States) equipped with a standard eight-channel phased-array head coil. Before scanning, patients wore earplugs to reduce the noise of the scanner. Sponge pads were fixed around all participants’ heads during scanning for minimize motion while maintaining the supine position. Every participant performed the following behaviors: keep quiet, stay awake, and keep eyes closed without thinking. All participants were checked by two experienced neuroradiologists to have no abnormalities on routine MRI.

High-resolution structural T1-weighted scan (Three-dimensional Brain Volume, 3D BRAVO) were performed with the following parameters: time repetition (TR) = 8.2 ms, time echo (TE) = 3.2 ms; flip angle = 12°, field of view (FOV) = 240 × 240 mm, slices = 115, voxel size = 1 mm, and thickness = 1.0 mm. Resting-state blood oxygenation level-dependent (BOLD) MR images were collected with the following parameters: TR = 2000 ms, TE = 30 ms, flip angle = 90°, FOV = 240 × 240 mm, matrix = 64 × 64, slice thickness = 4.0 mm, no interspace, the number of slices = 41, gradient echo-planar volumes = 200, and duration was 6 min 40 s.

### Processing of rs-fMRI data and ALFF analysis

Functional data preprocessing and statistical analyses were performed by using the Statistical Parametric Mapping 12 (SPM12, http://www.fil.ion.ucl.ac.uk/spm/) and the Resting-State fMRI Data Analysis Toolkit (REST 1.8, http://www.restfmri.net) on the MATLAB. The following steps include: the first 10 time-points of data were removed to eliminate the effects of inadaptability and magnetic field inhomogeneity. Then, slice timing step was performed, and rigid body-motion data correction steps was performed artificially removing data whose head-motion and rotation was >1.5 mm or >1.5°. The remaining dataset was spatially normalized to the MNI template. After normalization, all images were resampled into 3 × 3 × 3 mm^3^ and spatially smoothed with a 4 mm FWHM Gaussian kernel. The effects of high frequency signal, low frequency drift and physiological noise were removed by bandpass filtering (0.01–0.08 Hz) and linear drift. The ALFF analysis was calculated based on the Fast Fourier transform (FFT), the time series of each voxel was firstly converted to frequency domain, the square root was computed at each frequency of the power spectrum, and then the mean square root was obtained in the range of 0.01 to 0.08 Hz band for each voxel, finally, averaged square root was taken as ALFF.

### Statistical analysis

All data of internal tocodynamometry measurement were analyzed using commercial statistical package SPSS 23. Data were presented as frequencies and percentages for categorical variables and as mean (standard deviation) for numeric variables. The Chi-square (χ^2^) test was used to compare population distribution features between groups. The receiver operating characteristic (ROC) curve analysis was performed to determine the cut-off point for the magnitude of fetal head descending thrust at different cervical dilation distance to detect ECS, and the area under the ROC curve (AUC) was performed to determine the predictive value of various indicators ECS. To further verify the relationship between fetal head descending thrust and ECS, the Binary logistic regression model was estimated to determine the independent factors associated with ECS.

Demographic and clinical data was performed by SPSS 23. Two sample *t*-test were used to compare age, education level, family income, gestational weeks, body mass index, and PPQ/PCL-C/EPDS/PSQI scores between the PP-PTSD and HPW groups. The residential address, feeding and birth mode, living habit between the two groups were analyzed by the Chi-square (χ^2^) test. (Statistically significant correlation thresholds were set at a *p*-value of 0.05). The significant differences of ALFF value between the patients with PP-PTSD and HPW were analyzed by using REST software. Correction for multiple comparisons was performed by using a False discovery Rate (FDR) correction with *q*-value < 0.05.

The correlation analyses were performed by SPSS 23 between the ALFF value of the aberrant spontaneous brain activity region and the PPQ/PCL-C scores, exploring the association between functional abnormalities in PP-PTSD patients. (*p* < 0.05 was statistically significant).

## Results

### The internal tocodynamometry measurement results

The natural delivery rate of mothers with fetal head descending thrust <16.29 N (5–8 cm) were respectively lower than other mothers with fetal head descending thrust ≥16.29 N (5–8 cm), the natural delivery rate of mothers with fetal head descending thrust <26.36 N (8–10 cm) were respectively lower than other mothers with fetal head descending thrust ≥26.36 N (8–10 cm) (*P* < 0.01) (Table 1).

**Table 1:** The characteristics of internal tocodynamometry measurement results.

Degree of cervix dilation (cm)	3.00–5.00		5.00–8.00		8.00–10.00	
Mean value (N)	7.17		16.29		26.36	
Classification	*N* < 7.17	*N* ≥ 7.17	*N* < 16.29	*N* ≥ 16.29	*N* < 26.36	*N* ≥ 26.36
Standard deviation	6.84 ± 0.53	7.47 ± 0.48	14.23 ± 1.43	18.18 ± 0.96	24.58 ± 1.79	27.61 ± 1.32
Number of participants	115	130	117	128	101	144
Vaginal delivery (*n* = 186)	87 (75.65%)	99 (76.15%)	60 (51.28%)	126 (98.44%)	60 (59.41%)	126 (87.50%)
ECS (*n* = 59)	28 (24.35%)	31 (28.85%)	57 (48.72%)	2 (1.56%)	41 (40.59%)	18 (12.50%)
Chi-square value	0.01		74.35		25.63	
*P*-value	0.93		0.00		0.00	

**Fig. 3:**
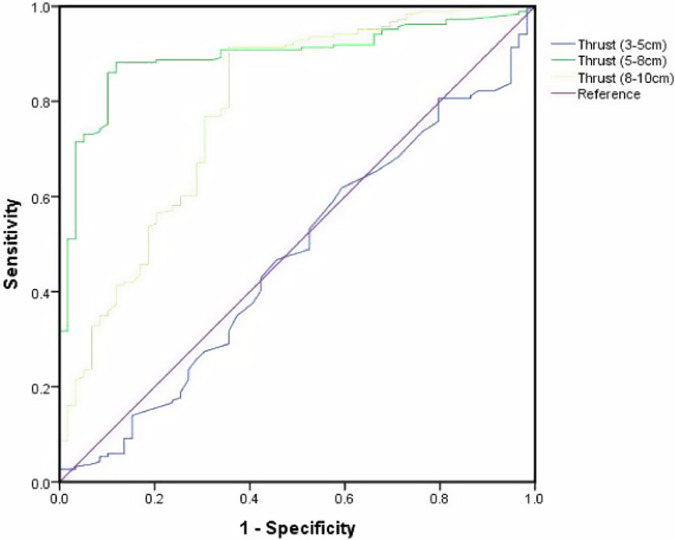
Receiver operating characteristic curve to assess the ability of different fetal head descending thrusts to predict emergency caesarean section. The blue curve was the thrust (3–5 cm), the green curve was the thrust (5–8 cm), the yellow curve was the thrust (8–10 cm) and the purple curve was the reference line. ROC receiver operating characteristic.

The ROC curve analysis results showed that the area under the receiver operating characteristic curve (AUC) value of thrust(5–8 cm) was 0.896 (95% CI: 0.854–0.938, *p* < 0.001), AUC of thrust(8–10 cm) was 0.786 (95% CI: 0.714–0.858, *p* < 0.001), which represented adequate performance for predicting ECS. The AUC of thrust(3–5 cm) was 0.472 (95% CI: 0.387–0.556, *p* = 0.515). (Table 2, Fig. 3)

**Table 2:** ROC curve to assess the ability of different fetal head descending thrusts to predict ECS.

Variables	AUC	SE	*p*	95%CI
Thrust(3–5 cm)	0.472	0.043	0.515	0.387–0.556
Thrust(5–8 cm)	0.896	0.022	0.000	0.854–0.938
Thrust(8–10 cm)	0.786	0.037	0.000	0.714–0.858

*AUC* the area under the receiver operating characteristic curve, *SE* Standard error.

Using the binary logistic regression analysis thrust (5–8 cm) and thrust(8–10 cm) were found to be independent correlates of emergency caesarean. Age and gestational weeks were not independently associated with ECS (Table 3).

**Table 3:** Binary logistic regression analysis of influencing factors of ECS.

Factors	B	Wals	Exp(B)	95% of CI	*p*-value
Age (years)	0.08	1.84	1.08	0.97–1.20	0.18
Gestational weeks	−0.17	1.08	0.84	0.61–1.17	0.30
Thrust (5–8 cm)	−0.85	40.83	0.43	0.33–0.55	0.00
Thrust (8–10 cm)	−0.74	27.64	0.48	0.36–0.63	0.00

### Demographic and clinical characteristics of PP-PTSD and HPW

The status of age, education level, family income, gestational weeks, body mass index, residential address, feeding mode, living habit showed no significant differences between the PP-PTSD and HPW groups (*p* > 0.05). The PP-PTSD group showed significant difference in PPQ/PCL-C/EPDS/PSQI score and birth mode than HPW (*p* < 0.05). (Table 4)

**Table 4:** Demographic and clinical characteristics of PP-PTSD patients and HPW.

Characteristics	PP-PTSD (*n* = 26)	HPW (*n* = 28)	*p*-value
Age (years)	27.54 ± 3.15	27.57 ± 3.51	0.97^a^
Education (years)	15.38 ± 1.65	15.54 ± 1.95	0.76^a^
Urban residents	18 (69.23%)	19 (67.86%)	0.91^b^
Family incomes	13.92 ± 2.88	13.71 ± 2.64	0.78^a^
Gestational weeks	39.59 ± 1.37	39.17 ± 1.47	0.28^a^
Breast feeding	24 (92.30%)	27 (96.42%)	0.51^b^
Caesarean	18 (69.23%)	8 (28.57%)	0.00^b^
Vaginal delivery	8 (30.77)	20 (71.43%)	0.00^b^
Smoking	2 (7.70%)	3 (10.71%)	0.70^b^
Drinking	3 (11.54%)	3 (10.71%)	0.92^b^
Body mass index (kg/m^2^)	23.15 ± 2.04	22.50 ± 2.29	0.27^a^
PPQ	28.27 ± 5.03	6.11 ± 1.77	0.00^a^
PCL-C	45.96 ± 4.40	10.00 ± 3.14	0.00^a^
EPDS	13.92 ± 2.24	0.54 ± 0.74	0.00^a^
PSQI	14.54 ± 3.24	5.21 ± 2.23	0.00^a^

*PP-PTSD* postpartum post-traumatic stress disorder, *HPW* healthy postpartum women, *PPQ* Perinatal Post-traumatic stress disorder Questionnaire, *PCL–C* Posttraumatic Stress Disorder Checklist–Civilian Version, *EPDS* the Edinburgh Postnatal Depression Scale, *PSQI* Postpartum Support Questionnaire. Family income, ten thousand RMB/year.

^a^independent-sample *t* tests.

^b^χ^2^, Chi-square.

### fMRI findings

**Fig. 4:**
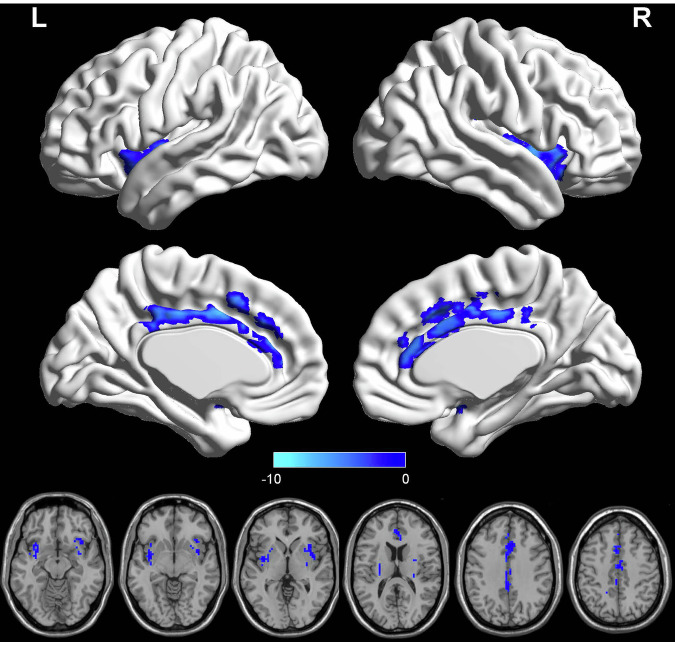
Compared with HPW, patients with PP-PTSD showed decreased ALFF in bilateral IC, right ACC and left MCC (FDR correction *q*-value  <  0.05). PP-PTSD postpartum post-traumatic stress disorder, HPW healthy postpartum women, IC insula cortex, ACC anterior cingulate cortex, MCC midcingulate cortex, ALFF amplitude of the low-frequency fluctuation.

At baseline, an initial group analysis was conducted on ALFF maps to explore the differences in regional activity between the PP-PTSD and HPW groups. The results indicated that PP-PTSD group showed decreased ALFF in the bilateral insula cortex (IC), right anterior cingulate cortex (ACC), left midcingulate cortex (MCC) compared with HPW (FDRcorrection *q*-value < 0.05) (Table 5, Fig. 4).

**Table 5:** Significant ALFF differences between PP-PTSD patients and HPW.

Location Cluster (AAL)	MNI					
	value	X	Y	Z	Cluster size (Voxels)	Peak intensity
Insula_R	decreased	39	3	−3	97	−7.51
Insula_L	decreased	−38	−6	−1	181	−6.43
Cingulum_Ant_R	decreased	3	33	24	94	−5.97
Cingulum_Mid_L	decreased	−3	3	39	221	−7.31

*AAL* Anatomical Automatic Labeling, *ALFF* the amplitude of the low-frequency fluctuation, *L* left, *R* right, *MNI* Montreal Neurological Institute.

### Correlation findings

**Fig. 5:**
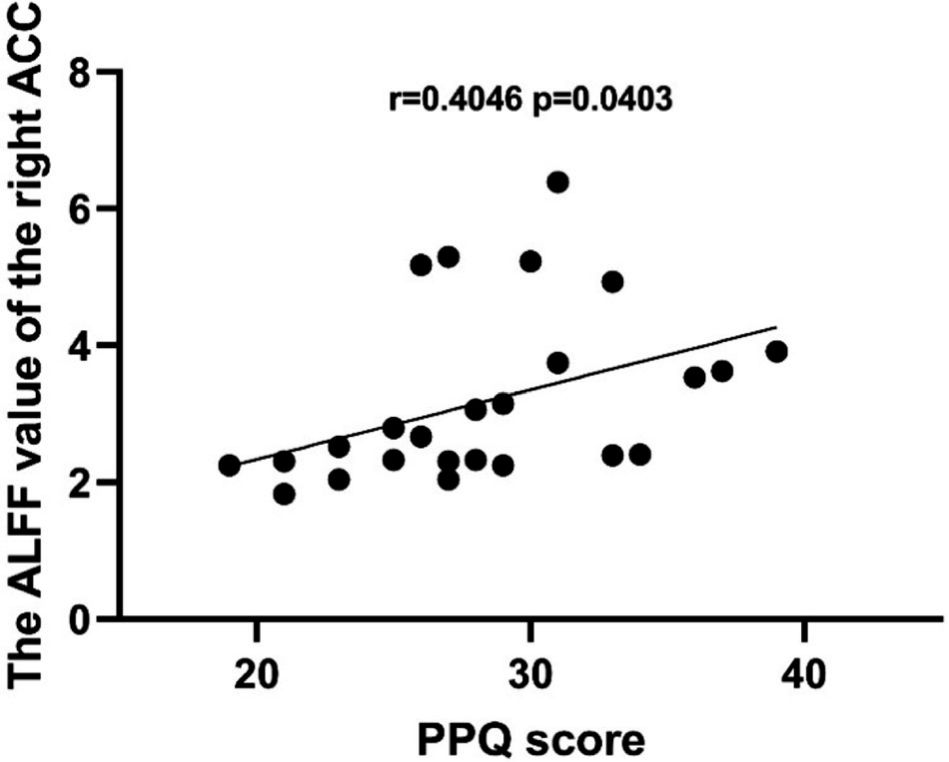
Correlation between PPQ score with the ALFF value of the right ACC. PPQ Perinatal Post-traumatic stress disorder Questionnaire, ACC anterior cingulate cortex, ALFF amplitude of the low-frequency fluctuation.

In PP-PTSD group, the ALFF value of the right ACC was positively correlated with PPQ score (r = 0.4046 *p* = 0.0403). The PPQ score showed no correlations with the ALFF value of the right IC (r = 0.3736 *p* = 0.0601), the left IC (r = 0.2580 *p* = 0.2032) and the left MCC (r = 0.2216. *p* = 0.2767) (Fig. 5).

**Fig. 6:**
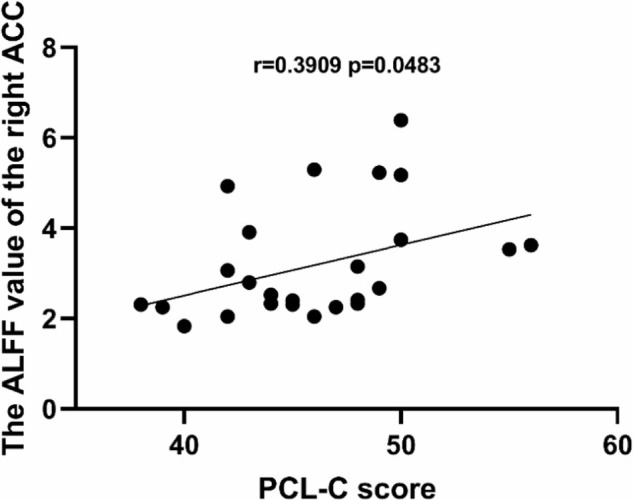
Correlation between PCL-C score with the ALFF value of the right ACC. PCL-C Post-traumatic Stress Disorder Checklist–Civilian Version, ACC anterior cingulate cortex, ALFF amplitude of the low-frequency fluctuation.

The ALFF value of the right ACC was positively correlated with PCL-C score (r = 0.3909 *p* = 0.0483). The PCL-C score showed no correlations with the ALFF value of the right IC (r = 0.1895 *p* = 0.3539), the left IC (r = 0.1962 *p* = 0.3366) and the left MCC (r = 0.2932. *p* = 0.1460) (Fig. 6).

## Discussion

The experiences of birth trauma can cause psychological stress symptoms up to PP-PTSD, with impact on women’s wellbeing. The mode of delivery has repeatedly been shown to be an important risk factor for PP-PTSD symptoms. In particular, a number of studies found an increased risk of PP-PTSD responses after ECS [24, 25]. From an obstetric point of view, a vaginal delivery is usually considered the best option in the absence of medical indications, because delivery by caesarean section is associated with increased infant and maternal risks, so that more mothers opted for vaginal delivery than elective caesarean delivery. But the mismatch between women’s preferred and actual mode of delivery would increase the risk of post-traumatic stress symptoms after childbirth [26]. ECS is a necessary choice in the process of labor, when parturient is unable to perform vaginal delivery due to abnormal uterine action. In this study, the internal tocodynamometry measurement was performed during labor for all mothers, and respectively taken at 3–5 cm, 5–8 cm, and 8–10 cm of cervical dilation. The ROC curve and binary logistic regression analyses were also performed to identify fetal head descending thrust that might help in the prediction of ECS. The rs-MRI was performed on 26 patients diagnosed with PP-PTSD of 245 mothers, ALFF technology was used to observe the spontaneous neural activity of all PP-PTSD patients, and correlation analyses were performed. Our results showed that the internal tocodynamometry measurement, might serve as basis for a potential predictive tool for ECS, and verified the presence of altered ALFF in the brain regions of PP-PTSD patients, mainly involving the bilateral IC, right ACC, and left MCC, that might be associated with emotion, cognition, and memory disorders functions in PP-PTSD patients.

Childbirth is a physiological process that women must go through after the end of pregnancy, which will be accompanied by severe pain and greater risk, painful memories of childbirth [27]. So childbirth is a very challenging event with one in three mothers experiencing birth trauma, characterized by extreme physical, emotional, or psychological distress [28]. Birth trauma could cause psychological distress, intense fear, or helplessness for the parturient and increases the risk of anxiety, depression and even PP-PTSD [29]. Birth trauma also has adverse implications for infant development and growth [30]. It has been reported that birth trauma was a robust and independent risk factor for PP-PTSD, above and beyond other known risk factors for PP-PTSD, and also a highly correlated with emergency cesarean section [31]. This study found that at different levels of cervical dilation (5–8 cm, 8–10 cm), there were different threshold levels of the fetal head descending thrust (<16.29/26.36 N), which were closely related to the occurrence of ECS and can be used as indicators to predict its occurrence, ROC curve and binary logistic regression analyses further verified the validity and reliability of the internal tocodynamometry measurement results. When the mother’s cervical dilation was opened to corresponding distance (5–8 cm/8–10 cm), but the fetal head descending thrust did not reach coincident threshold level of thrust (16.29/26.36 N), indicating that the mother might have to undergo ECS in a short time. ECS can cause terrible psychological and physical trauma to the mothers. Our study showed that internal tocodynamometry measurement may be used as a way to predict ECS, and reserve precious time for mothers to improve their psychological readiness for childbirth, so as to positively face possible emergencies during childbirth, and alleviate the fear of emergency situations. Implementing special midwifery-led emotional support programs can improve outcomes after a traumatic birth experience [32]. Giving continuous information and psychological support during childbirth can effectively reduce the occurrence of birth trauma, further to decrease the incidence of PP-PTSD.

At baseline, compared with HPW, analysis of the rs-fMRI data revealed decreased ALFF in the individuals with PP-PTSD in the right ACC, left MCC and bilateral IC, which manifested decreased spontaneous neuronal activity within the local brain region. The ACC and IC are critical parts of the salience network (SN), that plays an essential role in integrating internal and external stimuli, processes autonomic, interoceptive, homeostatic and cognitive information of personal relevance [33]. The ACC acts as a bridge between attentional and emotional processing, which is often described as a point of integration for visceral, attentional, and affective information that is critical for self-regulation and adaptability [34]. Lesions of the ACC produce striking personality changes and affective disorders, such as distress, anxiety, depression [35], insomnia as a risk factor for developing anxiety and depression [36], this may explain the sleep disturbances symptom in PP-PTSD patients. An fMRI study found reduced ALFF values in the ACC in patients with PTSD compared with healthy controls [37], and point out that ACC has long been considered to play a pivotal role in emotional processing, involved in negative emotion expression [38, 39]. This study showed that the ALFF value of ACC was positively correlated with PPQ and PCL-C scores, respectively, which may indicate that the functional deficits in the attention system and emotional cognitive dysregulation are related to the severity of clinical manifestations in PP-PTSD patients. The IC serves as an integrative hub between the sensory, interpretive and cognitive regions involved in emotion processing [40]. The IC provides an interface between bodily sensation and emotion, interacts with areas involved in cognitive and emotional control, and may have a key role in perceptual awareness, social behavior, and decision making [41]. The IC is considered to be associated with emotional response, especially in anxiety disorders [42]. We speculated that reduced reginal activity in the right ACC and bilateral IC may lead to SN network disorder, and patients with PP-PTSD cannot effectively integrate internal and external information, resulting in mutual control imbalance between emotional regulation and cognitive processing. One study reported that overgeneralization of contextual fear memory was a common symptom of PTSD, and this manifestation was closely related to the activity of the ACC [43]. Besides, IC is necessary for the acquisition and consolidation of contextual memory [44]. Therefore, our results may indicate a specific association between PP-PTSD and changes in memory functions, patients with PP-PTSD may suffer from long-term memory deficits. Intrusive memories can cause relive experiences of the traumatic event, which is diagnostic symptoms in patients with PP-PTSD, these intrusive memories may be easily triggered by ordinary stimuli such as the scream of infants, surgical pain caused by childbirth or anything that relive any aspect of the traumatic event [45, 46]. Converging evidence has linked the MCC to negative affect, pain and cognitive control, it has previously been proposed that this region uses information about punishment to control aversively motivated actions [47]. Abnormal activity of the left MCC may cause PP-PTSD patients to be unable to control aversive motivated behavior, such as thinking about birth trauma or even engaging in self-harming behavior.

The limitations of the present study are as follows: we investigated the relationship between the fetal head descending thrust and ECS outcomes of all participants, and observed abnormal regional activity changes in patients diagnosed with PP-PTSD. Firstly, these results should be interpreted cautiously, as the sample size of participants involved in internal tocodynamometry measurement and diagnosed PP-PTSD patients was small, and lack participants of other ethnic groups. We will continue to collect cases in the future to expand the sample size to validate the current findings. Secondly, although we ruled out most of the risk factors for PP-PTSD, there are still many factors that cannot be avoided, such as labor pain, anxiety before childbirth, concern for the newborn child, which are inevitable for mothers. Thirdly, elective caesarean delivery and vaginal birth may be responsible for the development of PP-PTSD, it can’t be ignored, although these risks of causing PP-PTSD is less than ECS. Fourthly, we invited two experienced senior psychiatrists to diagnose PP-PTSD in this study, and when two people had different views, we would invite another expert to participate in the evaluation. Finally, in addition to brain regional activity of PP-PTSD, the links between other brain functional connectivity, white matter, and gray matter morphology can be further explored, which may reveal more detailed information about PP-PTSD neuropathology by observing the relationship between structural and functional levels in the future.

## Conclusion

The present study provided the results of internal tocodynamometry measurement can serve as a predictive tool for ECS, on this basis, the implementation of effective emotional support may help to reduce the incidence of PP-PTSD. Besides, this study has verified the presence of altered spontaneous neuronal activity in the brain regions of PP-PTSD patients, mainly involving the right ACC, left MCC and bilateral IC. This study provided a protective prediction method to reduce the incidence of PP-PTSD, and also provided a new perspective to explore brain function abnormalities, increasing the understanding neurobiology of PP-PTSD.

## Data Availability

The data that support the findings of this study are available from the corresponding author, SZ and KL, upon reasonable request.
